# 2453: Serious cardiovascular morbidity and mortality in a cohort of adults with Fontan physiology

**DOI:** 10.1017/cts.2017.110

**Published:** 2018-05-10

**Authors:** Angela Weingarten, Daniel E. Clark, Ryan D. Byrne, Patricia Y. Chu, Frank A. Fish, Benjamin P. Frischhertz, Larry W. Markham

## Abstract

OBJECTIVES/SPECIFIC AIMS: The morbidity and mortality in adults with single ventricular hearts who have undergone Fontan palliation is poorly defined. These patients have a high burden of arrhythmia, heart failure, and re-operation. We hypothesized that age and type of Fontan predict occurrence of arrhythmia. METHODS/STUDY POPULATION: In total, 205 patients aged 18 years who had undergone a Fontan procedure were identified. Those with incomplete data were excluded. Demographic, anatomic, pharmacologic, imaging, hemodynamic, and electrophysiologic data were collected. The χ^2^ and Mann-Whitney *U* tests were used to test significance defined as *p*<0.05. RESULTS/ANTICIPATED RESULTS: Of the 205 patients identified, 59 had been lost to follow-up. Of the 146 patients (77, 53% female) actively followed 18 (12%) had died at a median (IQR) age of 27 (21–34.3); in patients alive as of 10/2016 the median age was 26 years (22–34). Fontan types were lateral tunnel (LT) (n=79, 54.1%), extracardiac (EC) (n=32, 22%), right atrial to pulmonary artery (RV-PA) (n=28, 19%), and Fontan with Bjork modification (n=4, 2.7%). Systemic left ventricle (n=96, 66%) was more common than systemic right ventricle (n=43, 30%). Of the 146 patients, 101 (69%) had significant morbidity or mortality: 86 (59%) were diagnosed with arrhythmia, 18 (12%) died, and 11 (8%) underwent heart transplants. Frequent procedures included: Fontan revisions/cryoablation in 28 (19%), electrophysiology studies with ablation in 73 (50%), and pacemakers in 53 (36%). Of the arrhythmia diagnoses, 57 (64%) were atrial tachyarrhythmias. RV-PA Fontan procedures were associated with significantly more atrial arrhythmia than all other Fontan types (70% vs. 30%; *p*<0.01). There was no statistical difference in occurrence of atrial arrhythmia in adults with LT Versus EC Fontans (*p*=0.3). While patients who had undergone RV-PA and Bjork Fontans were older with median age 34 years, there was no significant difference in age between LT and EC (median 24.0 and 24.5). DISCUSSION/SIGNIFICANCE OF IMPACT: Adult survivors of the Fontan procedure suffer from significant morbidity and mortality. The single most prevalent morbidity is atrial arrhythmia. We conclude that RV-PA Fontans, now obsolete, have the highest prevalence of arrhythmia and that there is no difference in arrhythmia burden between LT and EC Fontans. Given the high prevalence of morbidity and mortality in this population, it is imperative that they be followed by cardiologists with expertise in congenital heart disease.

